# Adverse Toxic Effects of Tyrosine Kinase Inhibitors on Non-Target Zebrafish Liver (ZFL) Cells

**DOI:** 10.3390/ijms24043894

**Published:** 2023-02-15

**Authors:** Katja Kološa, Bojana Žegura, Martina Štampar, Metka Filipič, Matjaž Novak

**Affiliations:** 1 Department of Genetic Toxicology and Cancer Biology, National Institute of Biology, Večna Pot 111, 1000 Ljubljana, Slovenia; 2 Jozef Stefan International Postgraduate School, 1000 Ljubljana, Slovenia

**Keywords:** zebrafish liver cells, ZFL, tyrosine kinase inhibitors, cytotoxicity, cell cycle, genotoxicity, environmental hazard

## Abstract

Over the past 20 years, numerous tyrosine kinase inhibitors (TKIs) have been introduced for targeted therapy of various types of malignancies. Due to frequent and increasing use, leading to eventual excretion with body fluids, their residues have been found in hospital and household wastewaters as well as surface water. However, the effects of TKI residues in the environment on aquatic organisms are poorly described. In the present study, we investigated the cytotoxic and genotoxic effects of five selected TKIs, namely erlotinib (ERL), dasatinib (DAS), nilotinib (NIL), regorafenib (REG), and sorafenib (SOR), using the in vitro zebrafish liver cell (ZFL) model. Cytotoxicity was determined using the MTS assay and propidium iodide (PI) live/dead staining by flow cytometry. DAS, SOR, and REG decreased ZFL cell viability dose- and time-dependently, with DAS being the most cytotoxic TKI studied. ERL and NIL did not affect viability at concentrations up to their maximum solubility; however, NIL was the only TKI that significantly decreased the proportion of PI negative cells as determined by the flow cytometry. Cell cycle progression analyses showed that DAS, ERL, REG, and SOR caused the cell cycle arrest of ZFL cells in the G0/G1 phase, with a concomitant decrease of cells in the S-phase fraction. No data could be obtained for NIL due to severe DNA fragmentation. The genotoxic activity of the investigated TKIs was evaluated using comet and cytokinesis block micronucleus (CBMN) assays. The dose-dependent induction of DNA single strand breaks was induced by NIL (≥2 μM), DAS (≥0.006 μM), and REG (≥0.8 μM), with DAS being the most potent. None of the TKIs studied induced micronuclei formation. These results suggest that normal non-target fish liver cells are sensitive to the TKIs studied in a concentration range similar to those previously reported for human cancer cell lines. Although the TKI concentrations that induced adverse effects in exposed ZFL cells are several orders of magnitude higher than those currently expected in the aquatic environment, the observed DNA damage and cell cycle effects suggest that residues of TKIs in the environment may pose a hazard to non-intentionally exposed organisms living in environments contaminated with TKIs.

## 1. Introduction

Scientific and public concern about the presence of pharmaceutical residues in the environment has increased as they are repeatedly detected worldwide, especially in aquatic environments. Of particular concern are cytotoxic anticancer drugs, as their therapeutic principle is based on damage to genetic material, which is associated with severe side effects in patients. Recently, residues of certain cytotoxic anticancer drugs have been reported to cause genotoxic effects in certain sensitive aquatic species, even at environmentally relevant concentrations [1].

Tyrosine kinase inhibitors (TKIs) are a group of drugs that have been increasingly used in the last 20 years for targeted therapies of various types of malignancies. Their main mechanism of action is binding to the active site of tyrosine kinases, thereby disabling the phosphorylation of tyrosine kinase secondary signal transducers involved in cell proliferation, growth, and angiogenesis, among others [2]. However, due to the conserved structure of the ATP-binding site, many TKIs exhibit inhibitory activity against a broad spectrum of protein kinases, which can affect multiple signaling pathways due to so-called off-target activities [3]. Moreover, signal transduction by tyrosine protein kinases is a general mechanism that is conserved across species, therefore off-target activities may also occur in other exposed non-target organisms in the environment [3].

The first drug in this class, imatinib mesylate (IM), was developed for the treatment of BCR-ABL associated leukemia and entered the market in 2001 [4]. It is still one of the most commonly used anticancer drugs in European countries [1]. Currently, 48 drugs targeting TKs have been approved for clinical use by the U.S. Food and Drug Administration, and many more are in clinical trials [5]. Therefore, it is expected that as the clinical use of TKIs increases, the occurrence of their residues in the aquatic environment will also increase each year. The analysis of the consumption of TKIs in Slovenia between 2010 and 2019 showed that the number of registered TKIs increased from nine in 2010 to thirty in 2019, and the total consumption of these drugs almost tripled from 42 kg/year to 115 kg/year [6]. Data on the occurrence in the environment are currently available, mainly for IM, which has been detected in hospital wastewater at concentrations of 164 ng/L and in the influent of wastewater treatment plants at concentrations of 11–577 ng/L [7]. In addition, ERL has been detected at a concentration of 3.9 ng/L in the Besòs River, Spain [8], and up to 8.1 ng/L in wastewater samples from Slovenia and Spain [9]. Meanwhile, for other selected TKIs, only predicted environmental concentrations (PEC) are available. PECs for SOR, REG, DAS, and NIL are 37.8 ng/L [10], 600 ng/L [11], 1800 ng/L [12], and 4000 ng/L [13], respectively. However, since the consumption of TKIs is constantly increasing and the PEC calculations are based on old consumption data, it can be assumed that TKI residues are now present in the environment in higher concentrations.

Ecotoxicological data for currently used TKIs are also sparse, with the exception of IM. Studies showed a high toxicity of IM to daphnids in chronic exposure [14,15], while algae [16], higher plants [17], and fish [18] were less sensitive. Moreover, the studies demonstrated that IM exerts genotoxic effects in daphnids [14] and higher plants [17], which was also confirmed in in vitro studies with fish and human cell lines [19,20,21]. The toxicological evaluation of IM, ERL, NIL, DAS, SOR, and REG on zebrafish (*Danio rerio*) embryos showed that the TKIs studied caused predominantly sublethal effects such as oedema, lack of blood circulation, and the formation of blood aggregates. The authors suggested that the observed sublethal effects are due to the antiangiogenic activity of the TKIs [6].

In this study, we applied the zebrafish liver (ZFL) cells as an experimental model to investigate the toxicity and potential genotoxicity of selected TKIs. Fish cell lines have been successfully used in the aquatic toxicological studies to investigate toxic and genotoxic effects, and can serve as an alternative to animal testing in preliminary eco-/toxicological studies [22]. Our previous studies have shown that ZFL cells are more sensitive and prone to detect primary DNA damage induced by certain anticancer drugs than human derived cell lines [20,21]. In the current study, we selected five TKIs (Table 1) that are most commonly used in cancer treatment and differ in their spectrum of target kinases. Potential genotoxicity was determined using the comet assay, which detects the induction of DNA strand breaks [23], and the micronucleus assay, which detects chromosomal damage [24]. As mentioned earlier, the mechanism of action of TKIs is to block the tyrosine kinase secondary signal transducers involved in cell proliferation and growth. To obtain additional information on the possible mechanisms involved in adverse effects of TKIs on non-target cells, their influence on the cell cycle distribution of ZFL cells was determined by flow cytometry.

## 2. Results and Discussion

Tyrosine kinase inhibitors (TKIs) have revolutionized the treatment of certain cancers by outperforming conventional (chemo) therapies in terms of selectivity, efficacy, and safety. As a result, their consumption is steadily increasing worldwide and their occurrence in the aquatic environment is expected to increase. As a consequence, non-target organisms in the environment can be exposed to TKI residues and may experience adverse effects. Unfortunately, experimental data on the potential genotoxicity of most TKIs to non-target organisms in the environment or environmentally relevant in vitro cell models are lacking, and existing studies are limited to human in vitro models, mostly on targeted cancer cell lines. Therefore, we aimed to investigate the cytotoxic and genotoxic effects of dasatinib (DAS), erlotinib (ERL), nilotinib (NIL), regorafenib (REG), and sorafenib (SOR) on zebrafish liver (ZFL) cells.

### 2.1. The Influence of TKI on the Viability of ZFL Cells

**Figure 1:**
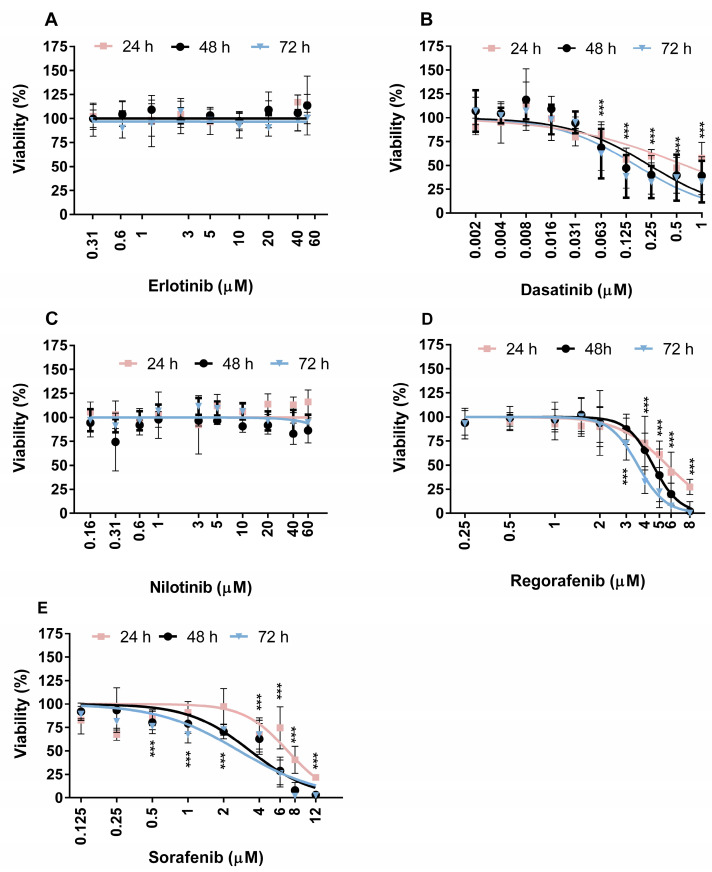
Effect of TKIs on the viability of zebrafish liver cells (ZFL): (**A**) erlotinib (ERL), (**B**) dasatinib (DAS), (**C**) nilotinib (NIL), (**D**) regorafenib (REG), (**E**) sorafenib (SOR). Values on graphs are presented using linear regression on an antilog numbering format of x axis. The viability of cells was determined with the MTS assay with three independent experiments of five replicates each. *** (*p* < 0.001).

The cytotoxicity of the selected TKIs was determined by MTS assay, and propidium iodide (PI) live/dead staining by flow cytometry. The viability of the ZFL cells was reduced after exposure to DAS, SOR, and REG in a dose- and time-dependent manner, while ERL and NIL did not affect cell viability at concentrations up to their maximum solubility (Figure 1A–E).

The calculated IC_50_ values after different exposure times showed comparable cytotoxic activity of SOR and REG (Table 2). DAS exerted significantly higher cytotoxic activity compared with SOR and REG, with more than 10-fold lower IC_50_ values (Table 2). Previously, Chang and Wang [32] reported that the IC_50_ value in nine human hepatocellular carcinoma cell lines exposed to DAS for 24 h ranged from 0.70 to 14.20 µM, while the IC_50_ values in human ovarian and breast cancer cell lines, exposed to DAS, ranged from 0.001 to 11.2 mM [32,33,34,35]. According to these data, ZFL cells are among the cells highly sensitive to DAS. Interestingly, DAS was shown to be cytotoxic only to primary rat hepatocytes, and not to primary human hepatocytes [36]. In primary rat hepatocytes, the 50% growth inhibition after 48 h exposure to DAS was at 21.1 μM and was shown to be due to the disruption of mitochondrial membrane potential and the activation of caspase cascade [37]. In the case of REG, a variety of human hepatocellular carcinoma cell lines (HuH-7, Hep3B, HepG2, Li-7) responded with significantly reduced cell proliferation after 48 h exposure to 3–5 μM REG [38,39]. Our experiments showed comparable sensitivity of ZFL cells (Table 2) with IC_50_ values of 5.64 μM, 4.54 μM, and 3.59 μM after 24, 48, and 72 h exposure, respectively.

Similar effects were induced by SOR, which blocks similar kinases to REG [29]. A significant change in cell viability was observed after 48 h exposure of Huh-7 and HepG2 cells to 2 μM and 5 μM SOR, respectively [39]. Furthermore, IC_50_ values of 10.04 μM and 5.46 μM were determined in primary human and rat hepatocytes, respectively, exposed to SOR for 24 h [40]. Our results demonstrate a comparable sensitivity of ZFL cells (Table 2) to SOR, with IC_50_ values of 6.93 μM, 3.36 μM, and 2.71 μM after 24, 48, and 72 h of incubation, respectively. In contrast, NIL and ERL showed no cytotoxic effects in ZFL cells at concentrations up to 60 µM for up to 72 h exposure. Similar to our results, ERL showed only slight cytotoxicity in HepG2 and HepaRG cells, where 20 µM ERL was the highest concentration tested due to its low solubility [41]. Moderate cytotoxicity of ERL was observed in target cell lines of non-small cell lung cancer H520 and H1975 cells, resulting in a 20% reduction in viability at the highest concentration tested [42]. In non-cancerous cell lines (murine macrophages RAW264.7, human keratinocytes HaCaT, rat astrocytes CTX TNA2, and human colon epithelial cells FHC), a low cytotoxicity of NIL at 40 µM was observed with a 5–20% reduction in cell viability [41]. In cancer cell models, Silveira et al. [28] showed that NIL at a concentration of 10 µM decreased the survival of target human adrenocortical carcinoma cells (H295R cells) by 62.9%. Chen et al. [43] reported the decreased viability of ovarian carcinoma cells (SKOV-3, A2780, ES -2 cells) in the range of 40–70% at NIL concentrations of 10 µM and 40 µM, respectively.

One of the markers of cytotoxicity, plasma membrane integrity, was further assessed using PI staining at low concentrations of the TKIs tested after 72 h of exposure, and a threshold of less than 25% decreased cell viability in the MTS assay was considered non-cytotoxic [44] and was used for further cell cycle analysis and genotoxicity testing.

**Figure 2:**
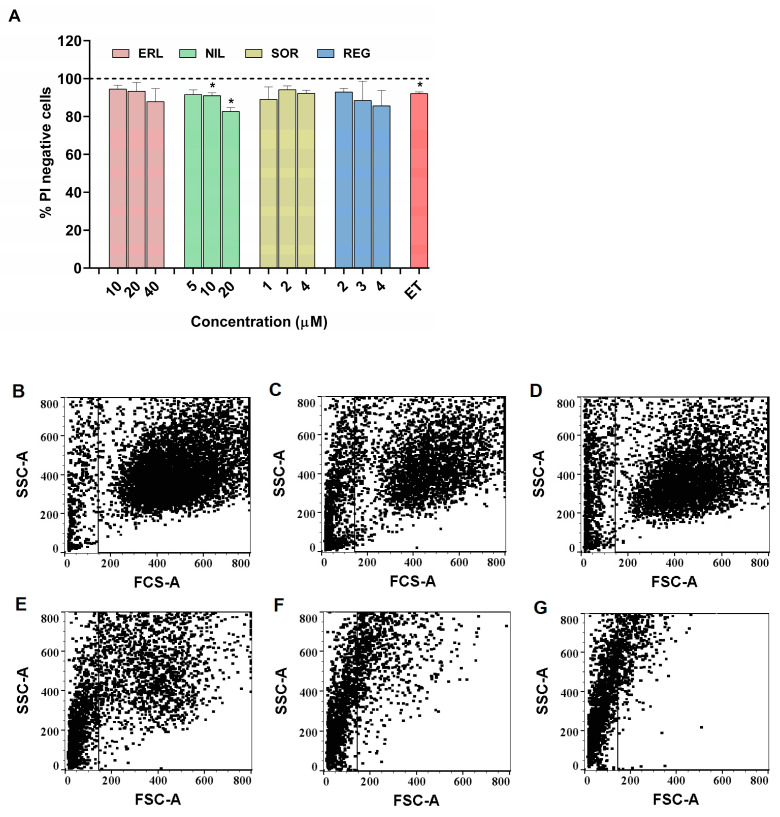
Effect of erlotinib (ERL), nilotinib (NIL), regorafenib (REG), and sorafenib (SOR) on zebrafish liver cell (ZFL) viability. (**A**) Viability of ZFL after 72 h exposure was determined using propidium iodide staining (PI) to distinguish between live and dead cells using flow cytometry. ET (0.17 μM) was used as a positive control. (**B**) Forward side scatter and side scatter (FSC/SSC) dot plots representing the distribution of ZFL cells after exposure to (**B**) control medium, (**C**) 0.17 μM etoposide, (**D**) 50 μM benzo[a]pirene, (**E**) 0.015 μM DAS, (**F**) 0.03 μM DAS, (**G**) 0.06 μM DAS. Three independent experiments were performed. * (*p* < 0.05).

ERL, SOR, and REG did not significantly decrease the percentage of PI negative cells, whereas NIL decreased the percentage of PI negative cells by approximately 20% at the highest concentration tested (Figure 2A). The increased number of PI positive/dead cells after exposure to NIL may indicate destabilized plasma membrane integrity, as no decrease in cell metabolic activity was observed with the MTS assay. In DAS exposed ZFL cells, the cell integrity was compromised even at the lowest DAS concentration (0.015 μM), and more than 50% of the exposed cells were degraded (Figure 2E–G). Moreover, at all tested DAS concentrations, we observed a shift of the exposed ZFL cells in the FSC/SSC dot plot to the upper left position (Figure 2E–G) compared to the control/untreated ZFL cells centered in the middle of the FSC/SSC dot plot (Figure 2B), which is an indicative parameter for cells undergoing the process of apoptosis [45]. These results indicate the induction of apoptosis in ZFL cells after exposure to DAS. DAS-induced apoptosis was confirmed in several hepatocellular carcinoma cell lines with an IC_50_ of 0.03–1 µM [33]. As reported by Mingard et al. [46], 20 μM DAS induced apoptosis in HepG2 cells, and a significant increase in the number of annexin V positive cells was detected along with the activation of caspase-3. Similar observations were made in normal rat hepatocytes, where 7.5 μM DAS induced membrane permeability and cell leakage in primary rat hepatocytes in vitro, while in in vivo experiments internucleosomal DNA fragmentation detected by DNA gel electrophoresis and activation of the caspase cascade by cleaved caspase-3 and cleaved PARP confirmed the onset of apoptosis [37].

### 2.2. The Influence of TKIs on Cell Cycle Progression of ZFL Cells

TKIs are homologs of adenosine triphosphate (ATP), which competitively occupy the ATP-binding site of protein tyrosine kinases (PTKs) and thus block PTK-mediated signaling pathways in cancer cells, thereby inhibiting their growth and proliferation [2]. Notwithstanding the wide variety of TKI targets, the suppression of cancer cell proliferation by blocking cell division in the G0/G1 phase of the cell cycle is one of the most prevalent anti-tumor mechanisms of numerous TKIs, including DAS, ERL, NIL, SOR, and REG [33,38,47,48,49,50].

**Figure 3:**
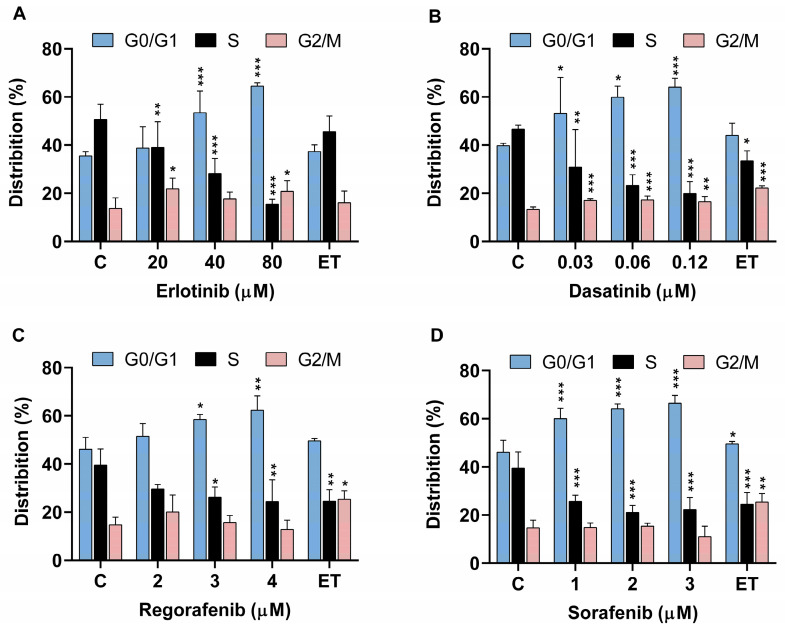
Flow cytometric analysis of cell cycle distribution of zebrafish liver cells (ZFL) after exposure to (**A**) erlotinib, (**B**) dasatinib, (**C**) regorafenib, (**D**) sorafenib for 72 h. ET (0.17 μM) was used as a positive control. Three independent experiments were performed. Significant difference between untreated control cells (**C**) and cells exposed to TKIs and ET. * (*p* < 0.05), ** (*p* < 0.01), *** (*p* < 0.001).

To date, there are no available in vitro studies investigating the effects of TKIs on the cell cycle progression of fish cell lines or other cells from aquatic organisms. In our study, the effects of selected TKIs on cell cycle progression were determined by flow cytometry. A concentration-dependent arrest of the cell cycle in the G0/G1 phase was found in parallel with a decrease in the S-phase fraction of the cells after treatment with DAS, ERL, REG, and SOR (Figure 3A–D). This is in line with several reports showing G0/G1 mediated cell cycle arrest in various human normal and cancer cell lines exposed to TKIs [33,38,47,48,49,50].

Cell cycle arrest in the G0/G1 phase and a concomitant reduction in the number of cells in the S-phase, as well as an induction of apoptosis, were confirmed in several hepatocellular carcinoma cell lines identified as more sensitive to DAS (IC_50_ 0.003–1 μM) [33]. Similarly, an accumulation of Huh-7 cells in the G0/G1 phase and a decrease in cell number in the S phase of the cell cycle were observed after exposure to 1.3 and 5 μM REG, respectively [38,48]. Moreover, the observed profile of cell cycle arrest in Huh-7 cells was also confirmed after ERL (25 μM) [51] and SOR (3.7 μM) exposure for 24 and 72 h, respectively [48]. SOR and DAS were found to arrest cells in the G0/G1 phase in a concentration range of 5.6–14.1 μM and 0.1–2.5 μM, respectively, in various human non-small cell lung carcinomas (NSCLC) [40,50,52,53]. Our results demonstrated the susceptibility of ZFL cells to the cell cycle blocking effect of the tested TKIs in a similar concentration range, as previously reported for hepatocellular carcinoma cell lines. Already, relatively low concentrations such as 0.03 μM DAS, 40 μM ERL, 3 μM REG, and 1 μM SOR induced G0/G1 cell cycle arrest in ZFL cells, which was accompanied by a decrease in the number of cells in S phase (Figure 3A–D). After the exposure of ZFL cells to NIL, insufficient data from a representative cell population were obtained for cell cycle analysis, possibly due to the detrimental effect of NIL on the plasma cell membrane, as previously discussed in the context of the PI cell viability experiments (Figure 2A). It should be noted that no cytotoxic effect of ERL was detected by the MTS assay at concentrations up to 60 μM after 72 h of treatment, while the cell cycle analysis showed G0/G1 phase arrest upon the exposure of ZFL cells to 40 μM and 80 μM ERL for 72 h. In the study by Paech et al. [41], low cytotoxicity but no change in ATP content was observed in HepG2 and HepaRG cells exposed to 20 μM ERL for up to 48 h. The authors concluded that the mechanism of ERL cytotoxicity was not related to a mitochondrial mechanism or impaired glycolysis, as was the case with other TKIs (IM, sunatinib, and lapatinib) tested in the same study. Consistent with our results, ERL induced cell cycle arrest (10 μM) and apoptosis (25 μM) in HepG2 after 24 h of exposure [51]. Based on these data, it can be concluded that due to the complexity of the EGFR signaling pathway network associated with cell proliferation, growth, and inhibition of apoptosis [25,54], multiple mechanisms may interplay in the observed G0/G1 cell cycle arrest in ZFL cells due to exposure to the EGFR inhibitor, ERL.

### 2.3. Induction of DNA Strand Breaks

**Figure 4:**
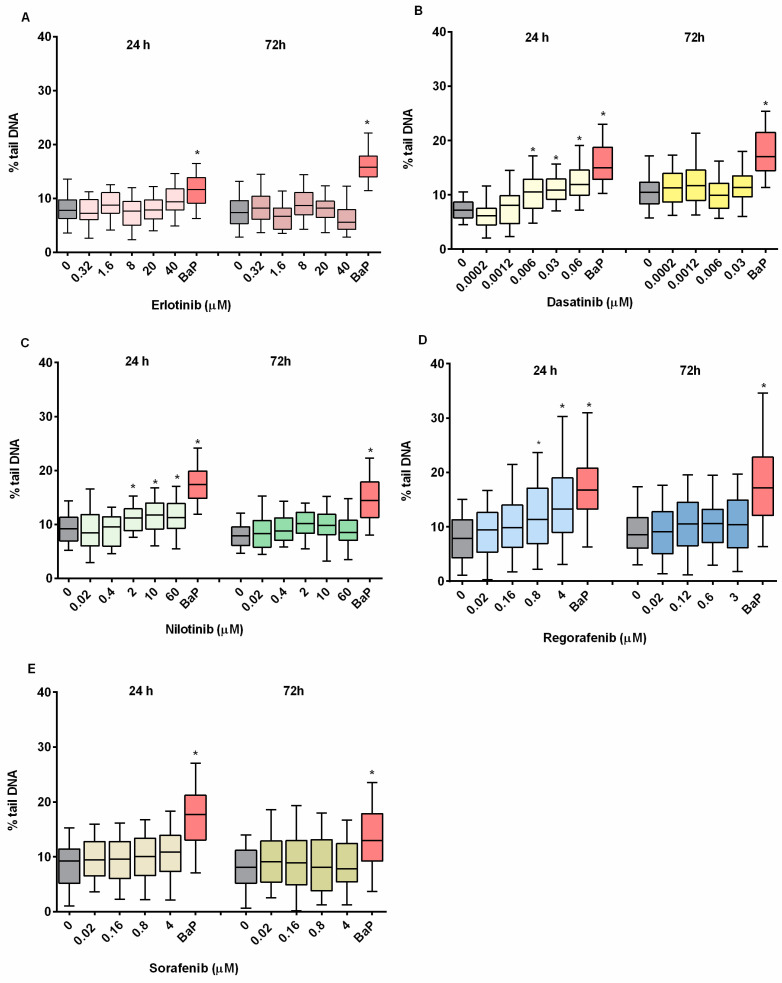
DNA damage evaluation in TKI-exposed ZFL cells. Cells were exposed to (**A**) erlotinib, (**B**) dasatinib, (**C**) nilotinib, (**D**) regorafenib, (**E**) sorafenib for 24 and 72 h. DNA damage was assessed by the comet assay and is expressed as percentage (%) of DNA in the comet tail. Fifty nuclei were measured per experimental point and are presented in box-plots. Three independent experiments were performed. B(a)P (benzo[a]pirene 50 µM) was used as positive control. * (*p* < 0.05).

The induction of DNA strand breaks was determined by the alkaline comet assay after the 24 and 72 h exposure of ZFL cells to non-cytotoxic concentrations of TKIs. A statistically significant increase in the percentage of tail DNA was observed in cells exposed to NIL (≥2 μM), DAS (≥0.006 μM), and REG (≥0.8 μM) after 24 h of exposure, with DAS being the most potent (Figure 4A–E). After 72 h of exposure, none of the TKIs studied induced DNA damage, suggesting that DNA strand breaks observed after 24 h exposure were most likely transiently present as intermediates formed during DNA repair processes. The division time of ZFL cells is approximately 72 h [55].

To our knowledge, there are no experimental data on the potential genotoxicity of the investigated TKIs to non-target organisms in the environment or environmentally relevant in vitro cell models. However, there are data on the genotoxicity of selected TKIs to human cells in vitro and mainly to targeted cancer cell lines. Several in vitro studies have confirmed that DAS [56,57] and REG [58,59] induce DNA damage assessed by comet assay in certain target cancer cells. In contrast to our results, the European Medicines Agency (EMEA) reported that NIL did not induce DNA single-strand breaks in the mouse L5178Y cell line evaluated by the comet assay [60]. Moreover, the exposure to SOR did not significantly increase DNA double strand breaks, as detected by the neutral comet assay in vitro in human colon adenocarcinoma HT-29 cell line treated with 16 uM for 12 h [61] and in HepG2 cells treated with 1 µM for 24 h, as detected by the alkaline comet assay [62]. In contrast, SOR induced DNA damage in vitro in Huh7 [62], A549 and MCF-7 [63] cells and in the liver of male albino rats treated with 10 mg/kg body weight daily for two weeks [64]. Diab et al. [64] indicated that DNA damage assessed by the comet assay in vivo was most likely of oxidative origin, as simultaneous treatment of male albino rats with SOR and antioxidants significantly reduced DNA damage induced by SOR. For ERL, Kryeziu et al. [65] showed that 20 µM ERL did not induce DNA damage (alkaline comet assay) in A549 cells after 6 h exposure [65]. Contrary to this and our study, Mak et al. [66] showed that ERL (10 µM) increases the percentage of tail DNA in human ovarian cancer cells (SKOV-3 cells), but only after a longer exposure time (72 h).

### 2.4. Induction of Genomic Instability

The induction of genomic instability after exposure to the TKIs investigated was determined by measuring micronuclei (MNi) with flow cytometry. Compared with the detection of micronuclei using the cytokinesis block micronucleus (CBMN) assay, the flow cytometric approach has several advantages, such as the ability to analyze a large number of cells and thus increase the statistical confidence of the results, the analysis being less time consuming, and the identification of micronuclei being much less subjective. Micronuclei are important biomarkers of chromosomal damage, genomic instability, and cancer risk. They are chromosome fragments or whole chromosomes that lag behind at anaphase during nuclear division [24]. Studies have shown that the increased percentages of micronuclei detected by flow cytometry and the classical CBMN method are comparable in vitro [67] and in vivo [68].

**Figure 5:**
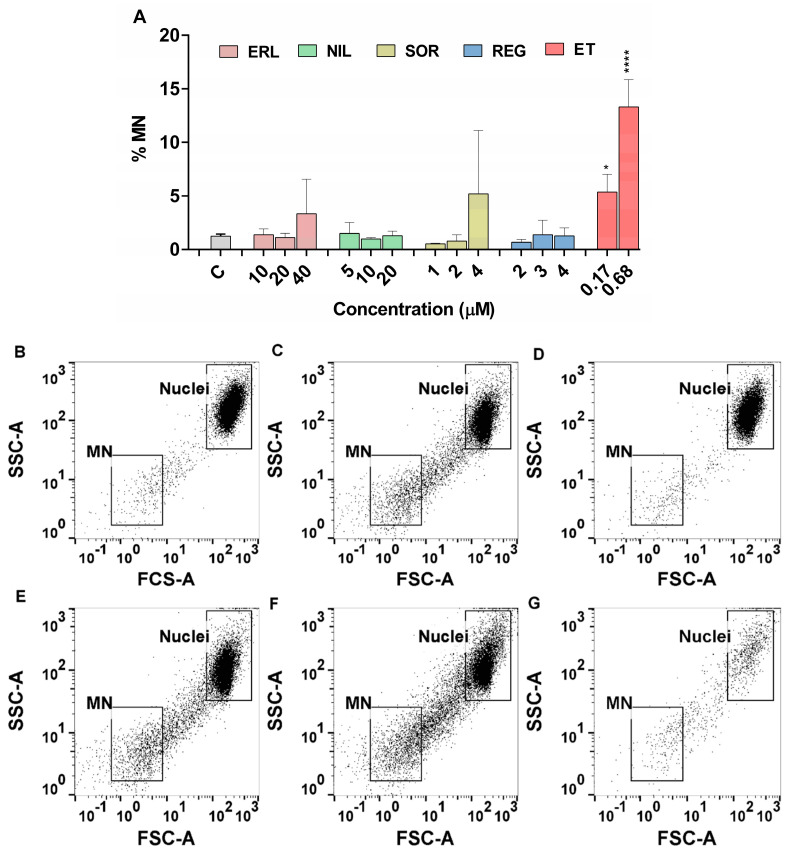
Induction of MN in ZFL cells after 72 h exposure to TKIs as determined by flow cytometry (**A**). A total of 20,000 nuclei were counted per each experimental point. Nuclei and MN were stained with Sytox Green, and dead cells were excluded from analysis using EMA (ethidium monoazide) stain. Three independent experiments were performed. ET (0.17 and 0.68 µM) was used as positive control. * (*p* < 0.05), **** (*p* < 0.0001). (**B**–**G**) Representative dot plots of ZFL nuclei and other subcellular particles as analyzed by flow cytometry. Dot plots show the distribution of nuclei (main nuclei population in upper right corner) and micronuclei particles (in lower left corner) within the forward scatter (FSC) vs. side scatter (SSC) region: (**B**) control (low amount of MNi); (**C**) 0.17 μM etoposide (increased amount of MNi); (**D**) 50 μM benzo[a]pirene (low amount of MNi); (**E**) 0.015 μM DAS (increased amount of MNi); (**F**) 0.03 μM DAS (increased amount of MNi); (**G**) 0.06 μM DAS (low amount of nuclei and MNi).

**Figure 6:**
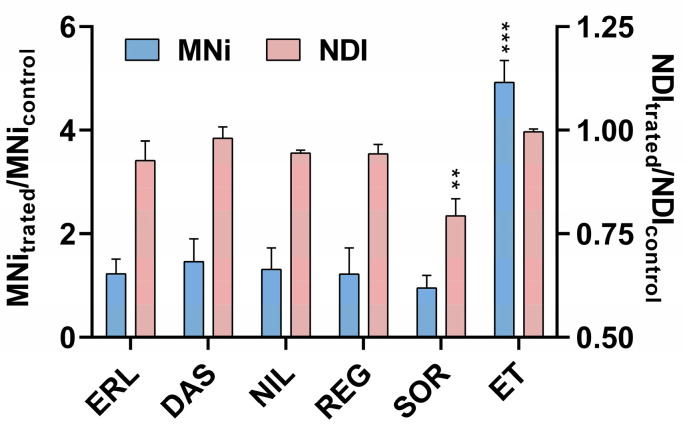
Induction of MN in ZFL cells after 72 h of exposure to TKIs as evaluated by cytokinesis-block micronucleus (CBMN) assay. The frequency of micronucleated (MNed) cells was determined in 1000 binucleated ZFL cells per replicate and experimental point. Etoposide (ET; 0.17 μM) was used as a positive control. The significant difference in the number of MNed cells between treated and control cells was determined with χ^2^ test ** *p* ˂ 0.001; *** *p* ˂ 0.0001. Data in the figure are presented as the ratio of MNi between the treated and control cells from three independent experiments ± SD.

The results of the present study showed that SOR, REG, NIL, and ERL did not significantly increase MNi frequency in ZFL cells at non-cytotoxic concentrations (Figure 5A). In cells exposed to DAS, MNi formation could not be accurately determined due to nuclear degradation. An increased signal for MNi population originating from degraded DNA was observed in DAS-treated cells (Figure 5E–G), but not in control (Figure 5B) or B(a)P treated cells (Figure 5D); however, some similarities were observed with MNi in ET-treated cells (Figure 5C). This can be explained by the results of PI staining, where the main population of cells was shifted to the upper left position of the FSC/SSC dot plot (Figure 2E–G), which is indicative of an apoptotic process and was further confirmed by the observed nuclear fragments in DAS-exposed cells (Figure 5E–G). The flow cytometry results were further confirmed by the CBMN assay, in which the TKIs, including DAS, were tested only at the highest concentration. None of the TKIs tested increased MNi formation, and with the exception of SOR, no decrease in the nuclear division index (NDI) was observed (Figure 6).

Our results are consistent with previous findings published by the European Medical Agency (EMEA), showing that REG [11], ERL [69], and NIL [59] are not mutagenic/genotoxic as they did not induce chromosomal damage/genomic instability in human lymphocytes or Chinese hamster ovary cells in vitro and in rat bone marrow. Moreover, REG (up to 4.4 μM) and ERL (up to 10 μM) after 24 h exposure did not induce MNi formation in human lymphoblastoid TK6 cells without and with metabolic activation [70] and ovarian carcinoma A2780 cells [71], respectively. ERL dose-dependently decreased the mitotic index and increased cytological aberrations in dividing and resting cells in the *Alium cepa* assay [72], which to the best of our knowledge is the only study using environmentally relevant organisms to assess chromosomal aberrations of selected TKIs. In contrast to our and the above-mentioned EMEA results, NIL at therapeutic concentrations increased the number of MNi with centromeric signals in targeted and non-targeted human chronic myeloid leukemia cancer cells after 48 h of treatment [73], suggesting aneugenic effects; however, no significant changes in telomere length and enzymatic telomerase activity were observed after treatment. In addition, NIL at therapeutic doses induced centrosome and chromosome aberrations, as well as spindle effects in normal human dermal fibroblasts treated for three weeks [47].

SOR and DAS increased the number of structural chromosomal aberrations in mammalian cells in an in vitro clastogenicity assay, but were negative in an in vivo micronucleus assay in mice and rats, respectively, as reported in EMEA documents (69). At therapeutic concentrations, DAS increased the number of centromere positive micronuclei in targeted and non-targeted human chronic myeloid leukemia cancer cells, but did not alter telomere length or telomerase enzymatic activity [73]. After 24 h of treatment, SOR at concentrations of approximately 5 μM and 10 μM did not increase MNi formation in exposed human hepatocellular carcinoma (SMMC-7721 and SK-HEP-1, respectively) cells [74], whereas it caused a strong (approximately 10-fold) increase in MNi formation in exposed Swiss albino mice [75].

Several authors suggested that the observed DNA damage or chromosomal aberrations could be the result of inhibition of one or more target or even non-target tyrosine kinases that regulate centrosome replication, DNA repair, and the cell cycle [47,76]. Several TKIs downregulate the expression of DNA repair proteins and genes, including Rad51 and MGMT, in various human cancer cell models in vitro [59,76,77,78,79], which is most likely a direct effect of tyrosine kinase inhibition. As a result, there may be impairments in DNA repair and even alterations in the ubiquitination of centrosomal components, leading to additional centrosomal duplication and mitotic spindle catastrophe [80]. NIL increased the protein expression of p53 and cleavage of poly (ADP-robse) polymerase (PARP) in vitro, suggesting potential DNA damage after treatment [81,82]. Interestingly, REG deregulated the expression of DNA damage-responsive genes *gadd45a* and *mcm6* in zebrafish embryos, suggesting that REG activates growth arrest signals in response to DNA damage [6].

## 3. Materials and Methods

### 3.1. Chemicals

The following tyrosine kinase inhibitors (TKIs) involved in the study were obtained as follows: dasatinib (DAS; MW 488.01; CAS 302962-49-8) from Santa Cruz Biotechnology (Santa Cruz, CA, USA), erlotinib (ERL; MW 393.4 CAS; 183321-74-6) from Apollo Scientific (Chesire, UK), nilotinib (NIL; MW 529.5; CAS 641571-10-0) from Sigma (St. Louis, MO, USA), regorafenib (REG; MW 482.8; CAS 755037-03-7) from Tokyo Chemical Industry Co (Tokyo, Japan), and sorafenib (SOR; MW 464.8 CAS; 284461-73-0) from Toronto Research Chemicals (Toronto, ON, Canada). Stock solutions were prepared in DMSO (Sigma Chemicals (St. Louis, MO, USA)): dasatinib (102.5 mM), erlotinib (58.2 mM), nilotinib (94.4 mM), regorafenib (103.6 mM), and sorafenib (107.6 mM). They were aliquoted, and stored at −20 °C.

Etoposide (ET; MW 588.6; CAS 33419-42-0) from Santa Cruz Biotechnology (Santa Cruz, CA, USA) and benzo(a)pyrene (B(a)P; MW 252.3; CAS 50-32-8) from Sigma (St. Louis, MO, USA) were used as positive controls. Stock solutions of etoposide (42.3 mM) and BaP (9.8 mM) were prepared sterile in DMSO, aliquoted, and stored at −20 °C.

Other chemicals were obtained as follows: HEPES and epidermal growth factor, Ethidium Monoazide Bromide (EMA), SYTOX™Green, Hoechst 33258 from Invitrogen (Carlsbad, CA, USA); 3-(4,5-dimethylthiazol-2-yl)-2,5-diphenyltetrazolium bromide (MTT), acridine orange (AO), cytohalasin-B, dimethyl sulfoxide (DMSO), ethidium bromide (EtBr), low-melting-point (LMP) agarose, normal-melting-point (NMP) agarose, methanol, sucrose from Sigma Chemicals (St. Louis, MO, USA); penicillin/streptomycin, L-glutamine, and phosphate-buffered saline (PBS) from PAA Laboratories (Dartmouth, MA, USA); Leibovitz L-15 medium and foetal bovine serum for ZFL cells from American Type Culture Collection (Manassas, VA, USA); Dulbecco’s modified Eagle’s medium and Ham’s F-12 medium from Gibco (Waltham, MA, USA); Trypsin-EDTA (0.25%) from Gibco, Life Technologies Corp., Carlsbad, CA, USA); Triton X-100 from Fisher Sciences (Waltham, MA, USA); citric acid, paraformaldehyde, ribonuclease inhibitor, sodium chloride, sodium citrate from Merck (Darmstadt, Germany).

### 3.2. Cell Culture

The ZFL cell line, obtained from American Type Culture Collection (N° CRL-2634), was derived from normal adult zebrafish [83]. Cells were grown under humidified air atmosphere at 28 °C in medium composed of 50% Leibovitz L-15 medium, 35% Dulbecco’s modified Eagle’s medium, and 15% HAM’S F-12 medium, supplemented with 5% heat-inactivated FBS; HEPES, 15 mM; NaHCO3, 0.15 g/L; insulin, 0.01 mg/mL; epidermal growth factor, 50 ng/mL, penicillin/streptomycin, 100 U/mL.

### 3.3. Determination of Cytotoxicity

#### 3.3.1. MTS Assay

The viability of ZFL cells after 24, 48, and 72 h of exposure to TKIs was determined using the tetrazolium-based (MTS) assay (Cell Titer 96 AQueous Non-Radioactive Cell Proliferation Assay; Promega, Madison, WI, USA) as previously described by Novak et al. [83]. Cells were seeded in 96-well microtiter plates (Corning Costar Corporation, New York, NY, USA) at a density of 7000 cells/well. After 24 h, the growth medium was replaced with fresh growth medium containing TKI at the following concentrations: dasatinib (0.002–1 μM), erlotinib (0.300–50 μM), nilotinib (0.156–60 μM), regorafenib (0.250–8 μM), and sorafenib (0.125–1 μM). Three independent experiments were performed, each with five replicates per treatment point. The statistical significance between the treated groups and the solvent control was determined by Student’s *t*-test using GraphPad Prism V6 (GraphPad Software, La Jolla, CA, USA). *** (*p* < 0.001)was considered statistically significant.

#### 3.3.2. Cell Viability Evaluation with Propidium Iodide by Flow Cytometry

Cells were seeded onto 24-well culture plates at a density of 35.000 cells/well for 24 h and then exposed to the TKIs studied: erlotinib (10, 20, and 40 μM), sorafenib (1, 2, and 4 μM), regorafenib (2, 3, and 4 μM), dasatinib (0.015, 0.03, and 0.06 μM), and nilotinib (5, 10, and 20 μM). Benzo[a]pyrene (50 μM), an indirect-acting genotoxic agent which requires metabolic activation for its genotoxic effects, and etoposide (0.17 μM), a direct-acting genotoxic agent, were selected as positive controls. After 72 h, floating and adherent cells were collected, resuspended in ice-cold 1x PBS, and immediately analyzed. Propidium iodide (PI) at a final concentration of 1 μg/mL was added to the cells 5 min before the start of measurements using the MACSQuant Analyzer 10 flow cytometer (Miltenyi Biotech, Bergisch Gladbach, Germany). MASCQuantifiy software (Miltenyi Biotech, Bergisch Gladbach, Germany) was used to quantify PI negative and positive cells. The experiments were repeated three times independently. Statistical significance between the treated groups and control was determined by one-way ANOVA with post hoc multiple comparison Dunett’s test using GraphPad Prism V6 (GraphPad Software, La Jolla, CA, USA). * *p* < 0.05 was considered statistically significant.

### 3.4. Cell Cycle Analysis with Flow Cytometry

ZFL cells were seeded onto T25 culture plates at a density of 450,000 cells for 24 h. Cells were then exposed to erlotinib (20, 40, and 60 μM), sorafenib (1, 2, and 4 μM), regorafenib (2, 3, and 4 μM), dasatinib (0.03, 0.06, and 0.12 μM), and nilotinib (5, 10, and 20 μM). The positive control was etoposide (0.17 μM), which is known to arrest cells in the G2/M phase of the cell cycle. After 72 h, cells were trypsinized (0.25% trypsin-EDTA) and washed with 1x PBS before fixation in 4% paraformaldehyde (PFA). For flow cytometric analysis, fixed cells were washed in cold 1x PBS and stained with Hoechst 33258 dye (diluted in 0.1% Triton X-100 1:500) as described by Stampar et al. [84]. Flow cytometric measurements were performed using a MACSQuant Analyzer 10 and the experiment was repeated three times independently. Each time, 20,000 single cells were recorded per experimental point. The data obtained were analyzed using FlowJo V10 software (Becton Dickinson, Franklin Lakes, NJ, USA). Statistical analysis for cell cycle was performed by the two-way ANOVA with Fisher’s LSD test using GraphPad Prism V6 (GraphPad Software, La Jolla, CA, USA). * *p* < 0.05, ** (*p* < 0.01), *** (*p* < 0.001) were considered statistically significant.

### 3.5. Determination of Genotoxicity

#### 3.5.1. Comet Assay

The induction of DNA strand breaks after TKI exposure was assessed by the comet assay according to Møller et al. [23], with minor modifications [20]. Briefly, ZFL cells were seeded at a density of 100,000 cells/well onto 12-well cell culture plates (Corning Costar Corporation, New York, NY, USA) and incubated for 24 h to attach. Cells were then treated with TKIs as follows: dasatinib (0.02–60 μM), erlotinib (0.32–40 μM), nilotinib (0.02–60 μM), regorafenib (0.02–4 μM), and sorafenib (0.02–4 μM) for 24 and 72 h, respectively. B(a)P (50 μM) was used as a positive control. After treatment, ZFL cells were trypsinized, collected, and centrifuged. Thirty μL of the cell suspension was mixed with 70 μL of 1% LMP agarose and placed on fully frosted slides pre-coated with 80 μL of 1% NMP agarose. Cells were then lysed for 1 h at 4 °C (2.5 M NaCl, 100 mM EDTA, 10 mM Tris, 1% Triton X-100, pH 10). DNA was subsequently denatured in electrophoresis buffer (1 mM EDTA, 300 mM NaOH, pH 13) for 20 min at 4 °C and slides were electrophoresed at 25 V (at 1 V/cm) for 20 min. Finally, nuclei were stained with GelRed (Biotium, Fremont, CA, USA). Images of 50 randomly selected nuclei per experimental point were acquired with the Eclipse 800 fluorescence microscope (Nikon, Tokyo, Japan) at 400× magnification using image analysis software (Comet Assay IV, Instem, UK). The statistical significance between control and treated groups was determined by one-way analysis of variance (ANOVA, Kruskal–Wallis) and Dunn’s multiple comparison test. * *p* < 0.05 was considered statistically significant.

#### 3.5.2. Cytockinesis-Block Micronucleus (CBMN) Assay

The CBMN cytome assay was performed according to Fenech [24] with minor modifications [20]. Briefly, 800,000 cells were plated onto 25 cm^2^ culture plates (Corning Costar Corporation, New York, NY, USA) and left for 24 h to attach. Cells were then treated for 72 h with TKIs at the following concentrations: dasatinib (0.03 μM), erlotinib (40 μM), nilotinib (20 μM), regorafenib (3 μM), and sorafenib (4 μM). ZFL cells were subsequently exposed to medium containing cytochalasin B (2 μg/mL) and incubated for an additional 48 h. The floating and adherent cells were then collected by trypsinization, centrifuged, washed with 1× PBS, and incubated in cold hypotonic solution (75 mM KCl) for 5 min. Fixed cells were transferred to a microscope slide, air dried, randomized, and coded. The slides were stained with AO (20 μg/mL) and analyzed at 400× magnification under a fluorescence microscope (Eclipse 800, Nikon, Japan). A total of 500 binucleated (BNC) cells (1000 BNC per replicate) with preserved cytoplasm were manually scored per experimental point. The number of micronuclei (MNi; expressed as the number of micronucleated (MNed) cells), nuclear buds (NBUDs) and nucleoplasmic bridges (NPBs) was determined according to standard criteria [85]. The nuclear division index (NDI) was estimated by scoring 500 cells with one to four nuclei. The NDI was calculated using the formula:

NDI = [M1 + 2M2 + 3(M3 + M4)]/500(1)

where M1, M2, M3, and M4 represent the number of cells with one to four nuclei, respectively [86]. Experiments were repeated three times independently. * *p* < 0.05 was considered statistically significant.

#### 3.5.3. Micronucleus Detection by Flow Cytometry

ZFL cells were seeded onto 24-well culture plates (Nunclon) at a density of 35,000 cells per well for 24 h. Cells were then treated with erlotinib (10, 20, and 40 μM), sorafenib (1, 2, and 4 μM), regorafenib (2, 3, and 4 μM), dasatinib (0.015, 0.03, and 0.06 μM), and nilotinib (5, 10, and 20 μM). Etoposide (0.17 μM and 0.68 μM) was used as a positive control. After 72 h, cells were placed on ice for 20 min, and subsequently a 0.05 mg/mL EMA solution was added to each well. The cells were placed on ice for 30 min and exposed to light for photoactivation of the EMA fluorochrome. Afterwards, cells were washed and resuspended in lysis solution (0.584 mg/mL NaCl, 1 mg/mL sodium citrate, and 0.3 μL/mL Triton X-100) containing 0.2 μM SYTOX Green and 0.25 mg/mL RNAse A, and incubated for 1 h in the dark at 37 °C. After incubation, lysis solution (85.6 mg/mL sucrose and 15 mg/mL citric acid) containing 0.2 μM SYTOX Green was added to the wells of the plate and incubated for a further 30 min at room temperature in the dark. Cells were analyzed on the same day using MACSQuant Analyzer 10 flow cytometer. MASCQuantify Software 2.11. was used to analyze each sample, and the percentage of micronuclei was determined after the acquisition of a total of 20,000 gated nuclei events, excluding EMA-positive events. One-way ANOVA with post hoc multiple comparison Dunett’s test using GraphPad Prism V6 (GraphPad Software, Carlsbad, CA, USA) was carried out. * *p* < 0.05 and *** *p* < 0.0001 were considered statistically significant.

## 4. Conclusions

In conclusion, the present study showed the cytotoxic effects of the investigated TKIs, as decreased ZFL cell viability and the arrest of ZFL cells in the G0/G1 phase of the cell cycle were confirmed. Nilotinib, dasatinib, and regorafenib induced the formation of DNA strand breaks, which were transiently present and most likely formed as intermediates in the DNA repair processes. However, none of the TKIs studied induced genomic instability of the ZFL cells. The results suggest that apoptosis may be involved in the mechanism of action of dasatinib. These results provide new insights into the potential adverse effects of TKIs on non-intentionally aquatic non-target organisms, as residues of TKIs in the environment may pose a risk to organisms living in these environments. Although the adverse effects have been observed at high concentrations of TKIs that are not currently expected to occur in the environment, a potential risk to unintentionally exposed environmental organisms cannot be completely excluded, particularly because of the increasing consumption of TKIs worldwide and the emergence of new TKIs in clinical practice, whose residues will eventually enter the environment.

## Figures and Tables

**Table 1:** Summary of tyrosine kinase inhibitors and their molecular targets.

Tyrosine Kinase Inhibitor	Tyrosine Kinase Targets	Reference
Erlotinib (ERL)	EGFR	[25]
Dasatinib (DAS)	SRC-family protein-tyrosine kinases/BCR-ABL kinases	[26]
Nilotinib (NIL)	Bcr-Abl tyrosine kinases/(*PDGF-R*) and c-kit	[27,28]
	cRaf1, BRaf, VEGFR, PDGFR	
Regorafenib (REG)	Angiogenic receptor tyrosine kinases (RTK) (VEGFR 1/3, TIE-2), oncogenic RTKs (*c-KIT, RET*), stromal RTKs (PDGFR-B, FGFR1), and intracellular signaling kinases (c-RAF/RAF-1, BRAF, BRAF)	[29,30]
Sorafenib (SOR)	Non-specific serine/threonine protein kinases	[31]
	(cRaf1, BRaf, VEGFR, PDGFR)	

**Table 2:** IC_50_ values of TKIs in zebrafish liver cells (ZFL) after 24, 48, and 72 h of exposure.

TKIs	Time of Exposure		
	IC_50_ for TKIs Cytotoxicity (95% Confidence Interval); μg/mL		
	24 h	48 h	72 h
Erlotinib (ERL)	ND	ND	ND
Dasatinib (DAS)	0.64	0.24	0.16
	(0.269 to 4.044)	(0.115 to 0.639)	(0.088 to 0.352)
Nilotinib (NIL)	ND	ND	ND
Sorafenib (SOR)	6.93	3.36	2.71
	(4.382 to 10.31)	(2.096 to 4.979)	(1.203 to 5.620)
Regorafenib (REG)	5.64	4.54	3.59
	(5.197 to 6.196)	(4.363 to 4.714)	(3.389 to 3.782)

ND, not determined.

## Data Availability

All the data supporting the reported results can be provided upon request to the corresponding authors.
